# Systemic oxidative stress levels and their associations with the risk of neovascular age-related macular degeneration and treatment response

**DOI:** 10.1186/s40942-025-00632-1

**Published:** 2025-02-10

**Authors:** Maiko Abe, Hiroshi Kunikata, Naoko Aizawa, Masayuki Yasuda, Fumihiko Nitta, Toshiaki Abe, Toru Nakazawa

**Affiliations:** 1 Department of Ophthalmology, Tohoku University Graduate School of Medicine, 1-1 Seiryo-machi, Aoba-ku, Sendai, Miyagi, 980-8574 Japan; 2 Department of Retinal Disease Control, Tohoku University Graduate School of Medicine, Sendai, Japan; 3 Division of Clinical Cell Therapy Center for Advanced Medical Research and Development, Tohoku University Graduate School of Medicine, Sendai, Japan; 4 Department of Ophthalmic Imaging and Information Analytics, Tohoku University Graduate School of Medicine, Sendai, Japan; 5 Department of Advanced Ophthalmic Medicine, Tohoku University Graduate School of Medicine, Sendai, Japan

**Keywords:** Systemic oxidative stress, Neovascular age-related macular degeneration, Treatment response, Advanced glycation end products, Skin autofluorescence

## Abstract

**Purpose:**

To investigate the association between oxidative stress (OS) and both the risk of neovascular age-related macular degeneration (nAMD) and the treatment response to intravitreal anti-vascular endothelial growth factor injections (anti-VEGF IVIs).

**Methods:**

This retrospective study included 46 treatment-naïve nAMD eyes of 46 patients (26 male and 20 female) who received anti-VEGF IVIs with a “treat-and-extend” regimen following an initial loading phase for one year. The patients were divided into two groups according to the total number of anti-VEGF IVIs administered during the year: the “effective” group and the “resistant” group. OS was evaluated by diacron reactive oxygen metabolites (d-ROMs), biological antioxidant potential (BAP), and skin autofluorescence (SAF) at baseline. For comparison, 54 control subjects were recruited.

**Results:**

There were no significant differences in d-ROM or BAP levels between control subjects and nAMD patients, regardless of sex, whereas SAF levels were higher in nAMD patients overall and in male nAMD patients than in controls (*P* < 0.001 for both). The effective and resistant groups included 30 and 16 eyes, respectively. Among the male nAMD patients, the effective and resistant groups had similar baseline characteristics, including age, smoking history, visual acuity, and central macular thickness; however, the resistant group had higher SAF levels (effective vs. resistant: 2.3 vs. 2.6 arbitrary units [AU]; *P* = 0.02). This finding was further supported by a multiple logistic regression analysis, which showed that the odds ratio for SAF was 1.57 per 0.1 AU increase (*P* = 0.01).

**Conclusion:**

SAF levels were significantly higher in nAMD patients than in controls. The total number of anti-VEGF IVIs required over one year in male nAMD patients depended on SAF levels, suggesting that the SAF levels may serve as a potential biomarker for the response to anti-VEGF IVIs in nAMD.

## Introduction

Neovascular age-related macular degeneration (nAMD) is the leading cause of blindness in developed countries [1, 2]. In recent years, intravitreal anti-vascular endothelial growth factor injections (anti-VEGF IVIs) has been the gold-standard treatment for nAMD, but the efficacy of this treatment and the required interval for the IVIs varies between individuals [3–6]. Although nAMD is a multifactorial disease and research has focused on oxidative stress (OS) as the most potent pathogenetic factor [7–9], the association between OS and clinical findings in nAMD remains unclear.

OS occurs when there is an imbalance between the production of reactive oxygen species (ROS) and the body’s ability to detoxify or repair the damage caused by these molecules. ROS are normal byproducts of cellular metabolism, but excessive accumulation of ROS can lead to oxidative damage in cells and tissues. In recent years, systemic OS has become easy to measure on an outpatient basis with techniques such as blood sampling, and its relationship with ocular diseases is becoming clearer [10–13]. OS parameters measurable in outpatient settings include diacron reactive oxygen metabolites (d-ROMs), biological antioxidant potential (BAP), and skin autofluorescence (SAF). d-ROMs serves as a biomarker of OS, measuring hydroperoxides, which are byproducts of ROS and lipid peroxidation. BAP assesses plasma antioxidant capacity, reflecting the body's ability to neutralize ROS and mitigate OS. SAF, which reflects the accumulation of advanced glycation end-products (AGEs) in tissues, is commonly used as a non-invasive indicator of AGE levels. AGEs, formed through non-enzymatic reactions of sugars with proteins, lipids, or DNA, promote OS and inflammation, which are associated with aging and chronic diseases such as diabetes and cardiovascular disorders.

Here, we investigate such systemic OS parameters in patients with nAMD and their associations with clinical findings, including the number of anti-VEGF IVIs.

## Methods

### Study design and participants

This was a retrospective study that examined 46 eyes of 46 nAMD patients (mean age: 77.0 ± 7.9 years, proportion of female patients: 43.5%) who visited Tohoku University Hospital, in Sendai, Japan. We also recruited 54 age- and gender-matched control subjects without nAMD (mean age: 74.9 ± 7.5 years, female ratio: 55.6%). This research followed the tenets of the Declaration of Helsinki and was approved by the Institutional Review Board of Tohoku University Graduate School of Medicine (2021-1-430). All subjects underwent a comprehensive eye screening that included a slit-lamp examination and a dilated biomicroscopic fundus examination. We obtained the history of smoking, diabetes mellitus (DM), and hypertension (HT) from medical records. nAMD was diagnosed by two experienced retina specialists (H.K. and M.Y.). Diagnosis and classification of nAMD were performed based on detailed ophthalmological examinations that included dilated fundus photography, optical coherence tomography (OCT; DRI swept-source OCT; Triton, Topcon, Japan), fluorescein angiography (FA), and/or indocyanine green angiography (ICG).

### Classification of treatment response

nAMD was classified into three subtypes based on OCT, FA and/or ICG findings: typical nAMD, polypoidal choroidal vasculopathy (PCV), and retinal angiomatous proliferation (RAP). We excluded patients with uveitis or diabetic retinopathy. We also excluded nAMD patients who had previously undergone anti-VEGF IVI treatment and those who received anti-VEGF drugs other than ranibizumab or aflibercept (ranibizumab 10 mg/mL [Lucentis; Novartis Pharma AG, Basel, Switzerland] or aflibercept 40 mg/mL [EYLEA; Bayer, Germany], respectively). Only patients for whom observations were available for at least 12 months after the IVIs were enrolled. The patients with nAMD received IVI treatment (ranibizumab or aflibercept) with a “treat and extend” (T&E) regimen after an initial phase during which they received one to three monthly IVIs, depending on disease activity. Disease activity was determined based on loss of VA and/or one of the following criteria: new hemorrhages in a fundus examination, and persistent or recurrent intraretinal fluid or serous retinal detachment (SRD) in OCT imaging. When a dry macula and no worsening of pigment epithelial detachment (PED) were documented, the treatment and follow-up visits were generally extended by periods of 4 weeks. The nAMD patients using the T&E regimen were classified into two groups based on the number of IVI treatments they received within 12 months, modified from a previous report using a “pro re nata” regimen [14]. The “effective” group received six or fewer IVIs, while the “resistant” group received seven or more IVIs. This classification is based on the criterion that if the treatment interval can be successfully extended after three loading doses, IVI treatments would occur six times over the course of one year.

### Measurements of oxidative stress biomarkers

d-ROM, BAP, and SAF levels were compared between the two nAMD groups, as well as between control subjects and nAMD patients. In principle, at the time of the first visit, d-ROM and BAP levels were measured with a free radical analyzer (Wismerll, Tokyo, Japan), and SAF levels were measured with an AGE Reader (DiagnOptics BV, Groningen, Netherlands). These methods are the same as those used in our previously published report [13]. Best-corrected visual acuity (VA) and central macular thickness (CMT) were measured at baseline and at each follow-up visit. All data are shown as the mean ± standard deviation.

### Statistics

The Mann–Whitney U test was used to compare continuous variables, and Fisher’s exact test was used to compare categorical variables between the groups. Separate multiple logistic regression analyses were performed to detect contributing factors to nAMD and resistant nAMD. We analyzed the male and female subgroups, as well as the overall group, for the following variables: smoking, typical nAMD, pre-treatment VA, pre-treatment CMT, and SAF. Receiver operating characteristic (ROC) curve analyses were also performed in the overall group and the male nAMD patients to assess the ability of the OS parameters (i.e., d-ROMs, BAP, and SAF levels) to predict nAMD in the overall group and resistant nAMD in the male nAMD patients, respectively. All statistical analyses were performed with Microsoft Excel 2019 (Microsoft, Redmond, WA, USA) or JMP software (Pro version 10.0.2, SAS Institute Japan Inc., Tokyo, Japan). Differences were considered significant at *P* < 0.05.

## Results

The overall characteristics of the nAMD patients and control subjects are shown in Table 1. The characteristics of the male nAMD patients and male control subjects are shown in Table 2, while those of the female nAMD patients and female control subjects are shown in Table. 3

**Table 1:** Overall characteristics of nAMD patients and control subjects

	Control	nAMD			*P* value	
		All	Effective	Resistant	C vs. nAMD	E vs. R
Number of cases	54	46	30	16	–	–
Age (years)	74.9 ± 7.5	77.0 ± 7.9	76.9 ± 8.6	77.1 ± 6.6	0.169	0.934
nAMD type (typical:PCV:RAP)	–	22:19:5	15: 2:3	7:7:2	–	0.914
Smoking (n, %)	16, 29.6	24, 52.2	16, 53.3	8, 50.0	0.023	0.829
HT (n, %)	30, 55.6	22, 47.8	18, 60.0	4, 25.0	0.446	0.009
DM (n, %)	4, 7.4	7, 15.2	6, 20.0	1, 6.2	0.230	0.216
d-ROMs (U. Carr)	387.6 ± 79.3	403.6 ± 70.6	393.8 ± 59.9	421.9 ± 86.5	0.290	0.259
BAP (μM/L)	2118.1 ± 212.6	2088.2 ± 289.2	2084.1 ± 229.5	2095.9 ± 386.0	0.564	0.912
SAF (AU)	2.0 ± 0.3	2.4 ± 0.5	2.3 ± 0.4	2.5 ± 0.5	< 0.001	0.159
Initial anti-VEGF drug (IVR:IVA)	–	18:28	13:17	5:11	–	0.424
Cases that switched drug (n, %)	–	17, 37.0	11, 36.7	6, 37.5	–	0.956
Number of IVIs (one year)	–	5.6 ± 1.7	4.8 ± 1.6	7.1 ± 0.3	–	< 0.001
VA (logMAR)						
Pre-treatment	–	0.40 ± 0.42	0.37 ± 0.41	0.47 ± 0.47	–	0.475
One month later	–	0.38 ± 0.43	0.33 ± 0.40	0.49 ± 0.50	–	0.287
One year later	–	0.36 ± 0.47	0.34 ± 0.51	0.40 ± 0.44	–	0.695
CMT (μm)						
Pre-treatment	–	342.0 ± 123.4	338.5 ± 134.9	348.5 ± 106.9	–	0.784
One month later	–	246.4 ± 74.9	238.7 ± 63.5	260.4 ± 94.8	–	0.419
One year later	–	245.3 ± 86.8	241.5 ± 66.1	252.3 ± 119.8	–	0.743

*nAMD* neovascular age-related macular degeneration, *PCV* polypoidal choroidal vasculopathy, *RAP* retinal angiomatous proliferation, *HT* hypertension, *DM* diabetes mellitus, *d-ROMs* derivatives of reactive oxygen metabolites, *U. Carr* Carrelli units, *BAP* biological antioxidant potential, *SAF* skin autofluorescence, *AU* arbitrary unit, *VEGF* vascular endothelial growth factor, *IVR* intravitreal ranibizumab, *IVA* intravitreal aflibercept, *IVIs* intravitreal injections, *CMT* central macular thickness, *VA* visual acuity, *logMAR* logarithmic minimum angle of resolution, *C* control, *E* effective, *R* resistant

**Table 2:** Characteristics of male nAMD patients and male control subjects

	Control	nAMD			*P* value	
		All	Effective	Resistant	C vs. nAMD	E vs. R
Number of cases	24	26	17	9	–	–
Age (years)	73.8 ± 6.8	76.5 ± 7.0	77.9 ± 7.0	73.9 ± 6.7	0.162	0.166
nAMD type (typical:PCV:RAP)	–	10:15:1	8:8:1	2:7:0	–	0.352
Smoking (n, %)	15, 62.5	22, 84.6	15, 88.2	7, 77.8	0.082	0.482
HT (n, %)	15, 62.5	14, 53.8	11, 64.7	3, 33.3	0.545	0.141
DM (n, %)	0, 0.00	5, 19.2	4, 23.5	1, 11.1	0.022	0.445
d-ROMs (U. Carr)	363.4 ± 63.9	390.3 ± 64.5	384.1 ± 58.0	401.9 ± 77.7	0.146	0.557
BAP (μM/L)	2115.8 ± 226.5	2132.2 ± 317.4	2128.5 ± 228.7	2139.1 ± 458.3	0.833	0.949
SAF (AU)	2.0 ± 0.2	2.4 ± 0.4	2.3 ± 0.4	2.6 ± 0.2	< 0.001	0.017
Initial anti-VEGF drug (IVR:IVA)	–	7:19	4:13	3:6	–	0.592
Cases that switched drug (n, %)	–	9, 34.6	5, 29.4	4, 44.4	–	0.443
Number of IVIs (one year)	–	5.5 ± 1.6	4.7 ± 1.5	7.0 ± 0.0	–	< 0.001
VA (logMAR)						
Pre-teatment	–	0.32 ± 0.38	0.28 ± 0.28	0.39 ± 0.54	–	0.573
One month later	–	0.34 ± 0.45	0.26 ± 0.31	0.49 ± 0.63	–	0.345
One year later	–	0.28 ± 0.40	0.26 ± 0.39	0.31 ± 0.46	–	0.786
CMT (μm)						
Pre-treatment	–	320.1 ± 66.6	308.4 ± 64.5	342.1 ± 72.7	–	0.261
One month later	–	243.3 ± 52.3	247.2 ± 48.6	236.4 ± 63.6	–	0.667
One year later	–	239.2 ± 76.7	241.6 ± 72.1	234.9 ± 92.7	–	0.854

*nAMD* neovascular age-related macular degeneration, *PCV* polypoidal choroidal vasculopathy, *RAP* retinal angiomatous proliferation, *HT* hypertension, *DM* diabetes mellitus, *d-ROMs* derivatives of reactive oxygen metabolites, *U. Carr* Carrelli units, *BAP* biological antioxidant potential, *SAF* skin autofluorescence, *AU* arbitrary unit, *VEGF* vascular endothelial growth factor, *IVR* intravitreal ranibizumab, *IVA* intravitreal aflibercept, *IVIs* intravitreal injections, *CMT* central macular thickness, *VA* visual acuity, *logMAR* logarithmic minimum angle of resolution, *C* control, *E* effective, *R* resistant

**Table 3:** Characteristics of female nAMD patients and female control subjects

	Control	nAMD			*P* value	
		All	Effective	Resistant	C vs. nAMD	E vs. R
Number of cases	30	20	13	7	–	–
Age (years)	75.7 ± 8.0	77.6 ± 9.1	75.6 ± 10.6	81.3 ± 3.6	0.459	0.099
nAMD type (typical:PCV:RAP)	–	12:4:4	7:4:2	5:0:2	–	0.501
Smoking (n, %)	1, 3.33	2, 0.10	1, 7.7	1, 14.3	0.391	0.639
HT (n, %)	15, 50.0	8, 20.0	7, 53.8	1, 14.3	0.497	0.013
DM (n, %)	4, 13.3	2, 10.0	2, 15.4	0, 0.0	0.723	0.274
d-ROMs (U. Carr)	407.0 ± 85.9	420.9 ± 76.1	406.5 ± 62.3	447.6 ± 96.3	0.552	0.336
BAP (μM/L)	2120.0 ± 204.7	2031.2 ± 243.9	2026.2 ± 225.9	2040.4 ± 293.4	0.187	0.913
SAF (AU)	2.1 ± 0.3	2.3 ± 0.6	2.3 ± 0.5	2.4 ± 0.7	0.076	0.742
Initial anti-VEGF drug (IVR:IVA)	–	11: 9	9: 4	2: 5	–	0.081
Cases that switched drug (n, %)	–	8, 40.0	6, 46.2	2, 28.6	–	0.444
Number of IVIs (one year)	–	5.8 ± 1.8	4.9 ± 1.8	7.3 ± 0.5	–	< 0.001
VA (logMAR)						
Pre-teatment	–	0.52 ± 0.45	0.49 ± 0.52	0.57 ± 0.37	–	0.683
One month later	–	0.43 ± 0.41	0.41 ± 0.49	0.49 ± 0.28	–	0.662
One year later	–	0.45 ± 0.54	0.43 ± 0.62	0.50 ± 0.42	–	0.771
CMT (μm)						
Pre-treatment	–	370.4 ± 166.8	377.8 ± 188.4	356.7 ± 146.2	–	0.786
One month later	–	250.3 ± 95.9	228.2 ± 79.1	291.3 ± 122.9	–	0.251
One year later	–	253.0 ± 97.4	241.3 ± 60.9	274.6 ± 153.0	–	0.598

*nAMD* neovascular age-related macular degeneration, *PCV* polypoidal choroidal vasculopathy, *RAP* retinal angiomatous proliferation, *HT* hypertension, *DM* diabetes mellitus, *d-ROMs* derivatives of reactive oxygen metabolites, *U. Carr* Carrelli units, *BAP* biological antioxidant potential, *SAF* skin autofluorescence, *AU* arbitrary unit, *VEGF* vascular endothelial growth factor, *IVR* intravitreal ranibizumab, *IVA* intravitreal aflibercept, *IVIs* intravitreal injections, *CMT* central macular thickness, *VA* visual acuity, *logMAR* logarithmic minimum angle of resolution, *C* control, *E* effective, *R* resistant

### Overall

Forty-six nAMD patients were enrolled in this study: 30 in the effective group and 16 in the resistant group (Table 1). We did not observe any significant differences in baseline characteristics, such as age, HT, and DM, between the control subjects and nAMD patients, but found a significant difference in smoking history (control: 29.6% vs. nAMD: 52.2%; *P* = 0.02). There were no significant differences in d-ROM or BAP levels between the control subjects and nAMD patients, but there was a significant difference in SAF (control: 2.0 AU vs. nAMD: 2.4 AU; *P* < 0.001). A multiple logistic regression analysis, with smoking history and SAF (per 0.1 AU increase) as explanatory variables, identified SAF as a significant contributing factor to nAMD (*P* < 0.001, odds ratio [OR]: 1.32, 95% confidence interval: 1.13–1.55), whereas smoking history was not significantly associated (*P* = 0.08). We did not observe any significant differences in baseline characteristics, such as age, nAMD subtype, smoking history, and DM, between the effective and resistant groups, but did observe a significant difference in HT incidence between the effective and resistant groups (effective: 60.0% vs. resistant: 25.0%; *P* = 0.01). There were no significant differences in d-ROM, BAP, or SAF levels between the effective and resistant groups. The effective and resistant groups showed no significant differences in VA or CMT at three time points: pre-treatment, after one month of treatment, and after one year of treatment. Furthermore, there were no significant sex differences in the three OS parameters, i.e., d-ROM, BAP, and SAF levels, in the overall group of patients and controls (*P* = 0.16*, P* = 0.23, and *P* = 0.44, respectively).

### Male

There were 17 and 9 male patients in the effective and resistant groups, respectively (Table 2). Among these male patients, no significant differences were observed between the control subjects and nAMD patients in baseline characteristics including age, smoking history, or HT, but there was a significant difference in DM (control: 0% vs. nAMD: 19.2%; *P* = 0.02). There were no significant differences in d-ROM or BAP levels between the control subjects and nAMD patients, but did show a significant difference in SAF levels (control: 2.0 AU vs. nAMD: 2.4 AU; *P* < 0.001). The effective and resistant groups showed no significant differences in age, nAMD subtype, smoking history, HT, or DM. There were no significant differences in d-ROM or BAP levels between the effective and resistant groups, but there was a significant difference in SAF levels (effective: 2.3 AU vs. resistant: 2.6 AU; *P* = 0.02). The effective and resistant groups also showed no significant differences in VA or CMT at three time points: pre-treatment, after one month of treatment, and after one year of treatment.

### Female

There were 13 and 7 female patients in the effective and resistant groups, respectively (Table 3). Among these female patients, no significant differences were observed between the control subjects and nAMD patients in baseline characteristics including age, nAMD subtype, smoking history, HT, and DM. There were no significant differences in d-ROM, BAP, or SAF levels between the control subjects and nAMD patients. No significant differences were observed between the effective and resistant groups in baseline characteristics including age, nAMD subtype, smoking history, and DM, but there was a significant difference in HT (effective: 53.8% vs. resistant: 14.3%; *P* = 0.01). The effective and resistant groups showed no significant differences in d-ROM, BAP, or SAF levels. The effective and resistant groups also showed no significant differences in VA or CMT at three time points: pre-treatment, after one month of treatment, and after one year of treatment.

### ROC

Table 4 shows the results of multiple logistic analyses to detect contributing factors to having resistant nAMD. We performed separate analyses of the overall group and the male and female subgroups with the following explanatory variables: smoking, typical nAMD, pre-treatment VA, pre-treatment CMT, and SAF. In the male subgroup, only SAF was confirmed as a contributing factor to resistant nAMD (OR for SAF: 1.57 per 0.1 AU increase; *P* = 0.01). No contributing factors were detected in the analyses of the overall group or female subgroup.

**Table 4:** Separate multiple logistic analyses of overall group and male and female subgroups to detect contributing factors to resistant nAMD

	Effective (n = 30)	Resistant (n = 16)	*P* value	OR (95% CI)
Overall (n = 46)				
Smoking (n, %)	16, 53.3	8, 50.0	0.734	0.78 (0.19–3.23)
Typical nAMD (n, %)	15, 50.0	7, 43.8	0.549	0.65 (0.16–2.63)
Pre-treatment VA (logMAR)	0.37 ± 0.41	0.47 ± 0.47	0.404	2.35 (0.31–17.95)
Pre-treatment CMT (μm)	338.5 ± 134.9	348.5 ± 106.9	0.874	1.00 (0.99–1.01)
SAF (0.1 AU increase)	2.3 ± 0.4	2.5 ± 0.5	0.490	1.06 (0.89–1.26)
	**(n = 17)**	**(n = 9)**		
Male (n = 26)				
Smoking (n, %)	15, 88.2	7, 77.8	0.196	0.07 (0.00–4.67)
Typical nAMD (n, %)	8, 47.1	2, 22.2	0.293	0.30 (0.03–3.14)
Pre-treatment VA (logMAR)	0.28 ± 0.28	0.39 ± 0.54	0.722	0.55 (0.02–14.14)
Pre-treatment CMT (μm)	308.4 ± 64.5	342.1 ± 72.7	0.188	1.01 (0.99–1.03)
SAF (0.1 AU increase)	2.3 ± 0.4	2.6 ± 0.2	0.013	1.57 (1.02–2.40)
	**(n = 13)**	**(n = 7)**		
Female (n = 20)				
Smoking (n, %)	1, 7.7	1, 14.3	0.630	2.16 (0.09–49.46)
Typical nAMD (n, %)	7, 53.8	5, 71.4	0.669	1.63 (0.17–15.58)
Pre-treatment VA (logMAR)	0.49 ± 0.52	0.57 ± 0.37	0.450	4.50 (0.08–245.17)
Pre-treatment CMT (μm)	377.8 ± 188.4	356.7 ± 146.2	0.405	1.00 (0.98–1.00)
SAF (0.1 AU increase)	2.3 ± 0.5	2.4 ± 0.7	0.874	1.01 (0.85–1.21)

*nAMD* neovascular age-related macular degeneration, *VA* visual acuity, *logMAR* logarithmic minimum angle of resolution, *CMT* central macular thickness, *SAF* skin autofluorescence, *AU* arbitrary unit, *OR* odds ratio, *CI* confidence interval

**Fig. 1:**
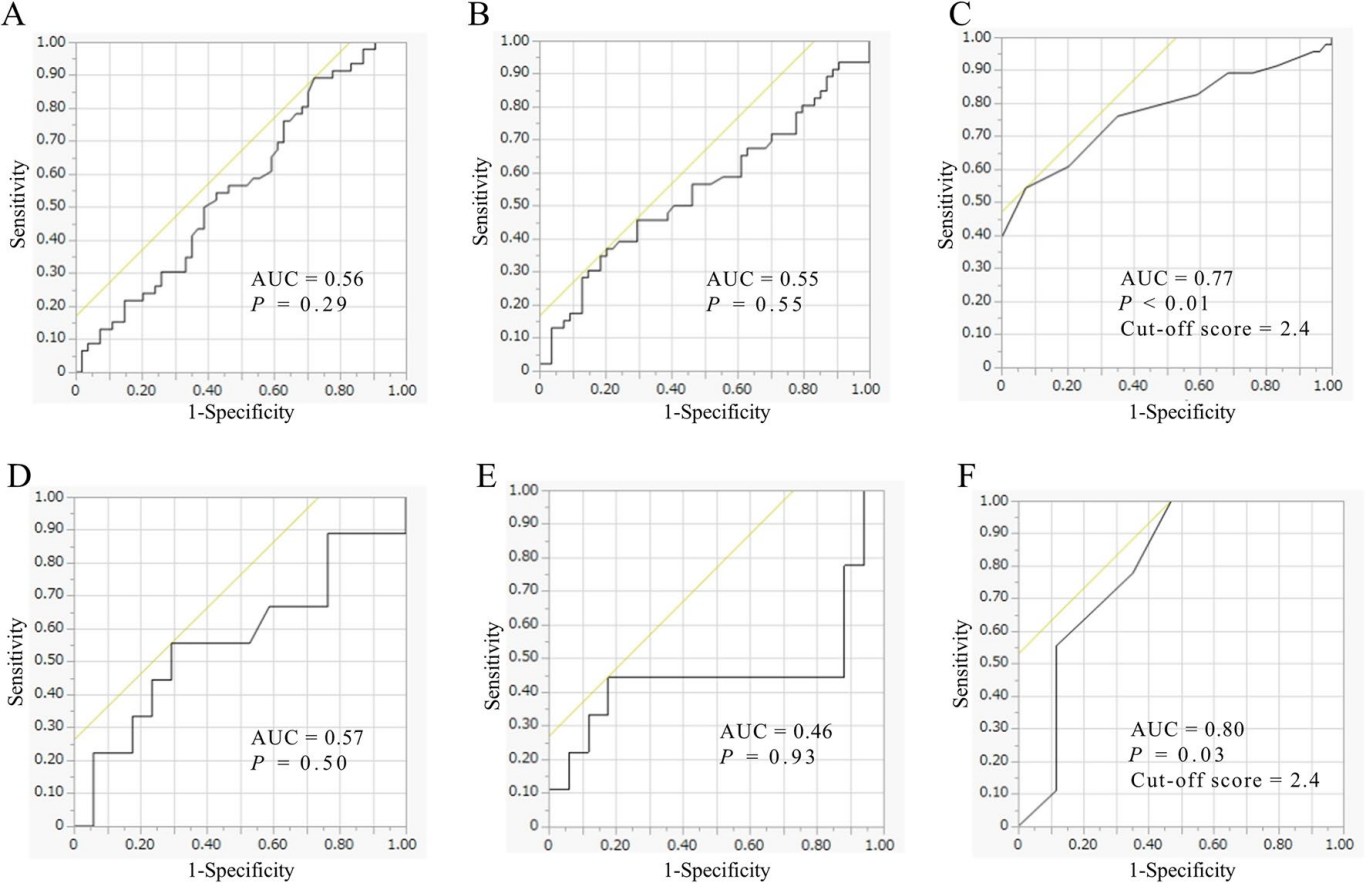
ROC curves based on OS parameters contributing to nAMD and resistant male nAMD. **A**, **B**, and **C** compare overall nAMD patients with control subjects; **D**, **E**, and **F** compare resistant male nAMD patients with effective male nAMD patients. **A** and **D** show d-ROM levels, **B** and **E** show BAP levels, and **C** and **F** show SAF levels

Figure 1 shows ROC curves based on the OS parameters for distinguishing nAMD patients from control subjects (Fig. 1A–C) and for distinguishing male patients with resistant nAMD from male patients with effective nAMD (Fig. 1D–F). d-ROM levels could not distinguish nAMD patients from control subjects (Fig. 1A: AUC = 0.56; *P* = 0.29). BAP levels could not distinguish nAMD patients from control subjects (Fig. 1B: AUC = 0.55; *P* = 0.55). SAF levels could distinguish nAMD patients from control subjects (Fig. 1C: AUC = 0.77; *P* < 0.01; cut-off score = 2.4 AU). d-ROM levels could not distinguish male patients with resistant nAMD from male patients with effective nAMD (Fig. 1D: AUC = 0.57; *P* = 0.50). BAP levels could not distinguish male patients with resistant nAMD from male patients with effective nAMD (Fig. 1E: AUC = 0.46; *P* = 0.93). SAF levels could distinguish male patients with resistant nAMD from male patients with effective nAMD (Fig. 1F: AUC = 0.80; *P* = 0.03; cut-off score = 2.4 AU).

**Fig. 2:**
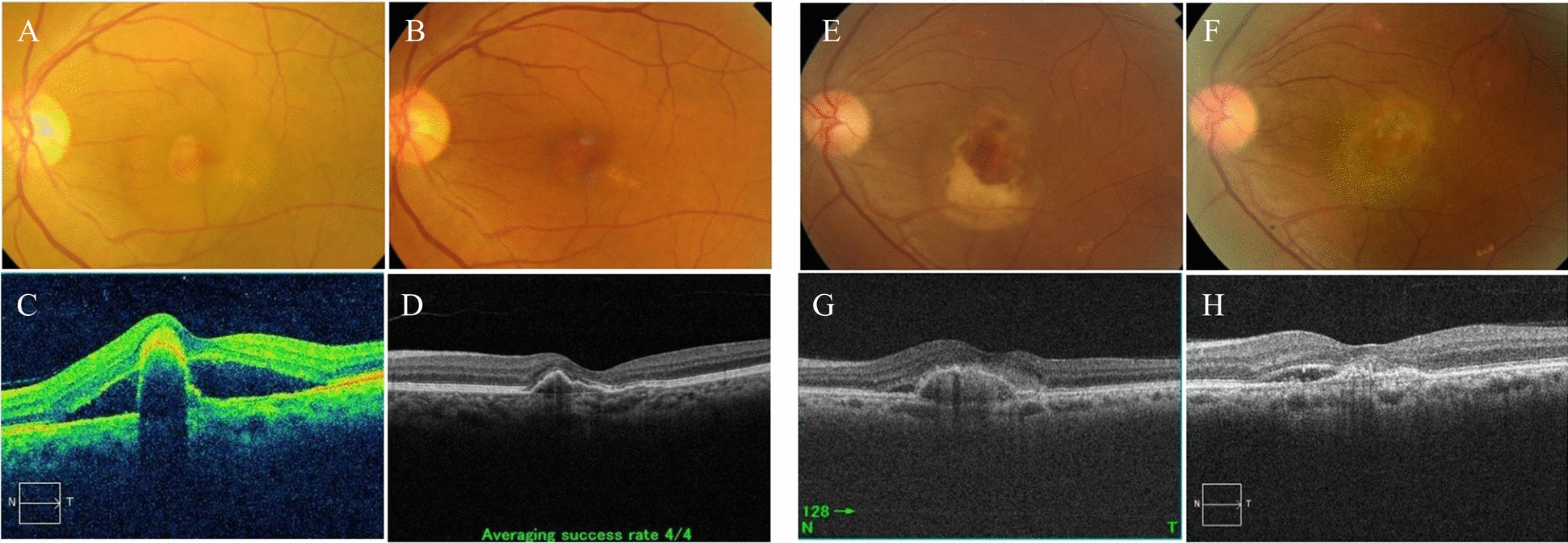
Fundus photographs and OCT images of typical cases with effective and resistant nAMD before and one year after treatment. **A**–**D** show a 68-year-old man with effective nAMD; **E**–**H** show a 58-year-old man with resistant nAMD. **A**–**D** The 68-year-old man with PCV in the left eye underwent six IVI treatments in one year. VA improved from 0.16 logMAR pre-treatment to 0 logMAR one year after treatment. Pre-treatment SAF level was 2.1 AU. **A** Fundus photograph pre-treatment showing a small subretinal hemorrhage and orange-red lesions in the fovea, surrounded by SRD. **B** Fundus photograph one year after treatment showing no SRD. **C** OCT image pre-treatment showing high, small PED surrounded by SRD. **D** OCT image one year after treatment showing reduced PED with no SRD. **E**–**H** The 58-year-old man with PCV in the left eye underwent seven IVI treatments in one year. VA changed from 0.22 logMAR to 0.16 logMAR one year after treatment, but no meaningful change was observed. Pre-treatment SAF level was 2.9 AU. **E** Fundus photograph pre-treatment showing a moderate subretinal hemorrhage and orange-red lesions in the fovea, surrounded by SRD. **F** Fundus photograph one year after treatment showing no hemorrhage; a white atrophic region was present in the fovea. **G** OCT image pre-treatment showing a low, small PED surrounded by SRD. **H** OCT image one year after treatment showing no remarkable change in foveal morphology; the PED reduced but the SRD remained

Figure 2 shows fundus photographs and OCT images of representative cases with effective and resistant nAMD.

## Discussion

In this study, we found that SAF levels were higher in patients with nAMD overall compared to controls. Additionally, SAF levels were higher in resistant male nAMD patients compared to effective male nAMD patients, which was further supported by a multiple logistic regression analysis.

### OS as a contributing factor to nAMD

The current study confirmed that higher SAF, an indicator of accumulated AGEs, was closely associated with presence of nAMD (AUC: 0.77; cut-off score: 2.4 AU). Systemic OS plays a role in the development and progression of nAMD, similar to its role in glaucoma [7]. Accumulation of ROS and oxidative damage over time can result from lipid peroxidation, DNA and protein damage, inflammation, and impaired antioxidant defenses, all of which contribute to the pathogenesis of nAMD [8, 9]. While there have been few clinical studies measuring systemic OS in patients with nAMD, our findings align with reports showing high SAF and low BAP levels in related conditions such as central serous chorioretinopathy, which shares some genetic factors with nAMD [13, 15]. The observed increase in SAF levels in nAMD patients aligns with the known pro-oxidant and pro-inflammatory roles of AGEs in promoting OS, inflammation, chemotaxis, and neovascularization in the retinal pigment epithelium, which are associated with the development of nAMD [16]. While no significant differences in d-ROM or BAP levels were observed between nAMD patients and controls in the current study, a previous report noted significantly higher d-ROM levels in male nAMD patients compared to male controls [17]. That study did not examine SAF levels but did examine BAP levels; the findings showed that there was no significant differences in BAP levels between nAMD patients and controls among either sex. These findings, combined with our results, suggest that while gender-specific variations may exist, systemic OS appears to be involved in nAMD. To reduce OS-related cell damage and prevent the onset and progression of nAMD, antioxidant therapies, such as supplements containing food-derived antioxidants, might contribute to this goal as part of a broader therapeutic approach [18, 19]. Further investigation is needed to identify systemic OS parameters with the greatest clinical relevance to nAMD.

### Sex differences in OS and its association with clinical outcomes in nAMD

Our results from the analysis of the male subgroup showed that male patients with resistant nAMD had higher SAF levels than male patients with effective nAMD (AUC: 0.80; cut-off score: 2.4 AU); however, we did not observe this association in the female patients. Although OS has been implicated in the pathogenesis of many ocular diseases, gender differences in associations between OS parameters and clinical findings have only recently been reported. In one report, lower BAP was associated with reduced blood flow in the optic nerve head only in male glaucoma patients [20]. Additionally, BAP was reported to be an independent contributing factor to a weighted estimate of the number of surviving retinal ganglion cells in young male glaucoma patients [21]. Furthermore, another study reported a significant correlation between d-ROM levels and the area of the choroidal neovascularization lesions in male nAMD patients [17]. Thus, male patients may be more susceptible to the harmful effects of exposure to OS, and this may contribute to the association with clinical findings in various ocular diseases, including nAMD.

Although our study found that there were no significant differences between male and female nAMD patients in the three OS parameters we examined, i.e., d-ROM, BAP, and SAF levels, the proportion of resistant nAMD patients was also similar between male and female patients (34.6% and 35.0%, respectively). However, male patients with resistant nAMD had significantly higher SAF levels than male patients with effective nAMD. Sex-related differences in the association between SAF levels and anti-VEGF IVI treatment number might be related to female hormones such as estradiol and estrogen, which may have a protective effect against neurodegenerative diseases, most likely via activation of the antioxidant defense system [22–24]. Estrogen has antioxidant properties and can scavenge free radicals, while the role of testosterone, a male hormone, in either promoting or inhibiting OS-induced cell damage remains a topic of controversy [25–27]. Several other factors, including enzymes and lifestyle and genetic factors, may account for sex differences in the impact of OS. These differences also have implications for susceptibility to nAMD and treatment outcomes. However, it is crucial to recognize that sex differences in OS are complex and can vary depending on the population and disease condition under study.

### Other factors affecting clinical outcomes in nAMD

Several factors, including the subtype of nAMD, disease severity, and VA, can influence clinical outcomes and treatment intervals in nAMD. Baseline VA at the initiation of anti-VEGF treatment is also a key factor in determining outcomes [28–30]. Patients with better initial VA often achieve more favorable prognoses and require fewer treatments compared to those with poorer baseline VA. Furthermore, OCTA characteristics, including features of neovascularization and the choroid, offer valuable insights into treatment needs, recurrence risk, and functional outcomes [14, 31, 32]. However, systemic factors are increasingly recognized as critical determinants of prognosis. Coexisting health conditions, including genetic predispositions [33–35], cardiovascular disease [36], diabetes [35], and hypertension [35, 37], have been reported to influence nAMD outcomes. This may be because factors such as systemic inflammation are thought to be involved in the progression and prognosis of nAMD [38–43]. These systemic factors may interact with ocular disease mechanisms and influence the response to anti-VEGF therapy, which in turn may affect the frequency of IVI treatments required. Considering the overall health status and systemic risk factors is essential for optimizing treatment strategies and improving clinical outcomes in nAMD patients.

### Limitations

The current study was somewhat limited by its retrospective design and the small number of patients, all of whom were Japanese. The frequency of anti-VEGF IVIs may vary by drug type, and ideally, analysis should be conducted separately for each drug; however, this was not feasible due to the limited number of cases. There was also a significant difference in the smoking rate between the control subjects and nAMD patients among the overall group. This is understandable, as smoking could increase OS and SAF levels, whose changes may be associated with nAMD. Additionally, although there were no significant differences in the rate of HT between the control subjects and nAMD patients in both the overall group and the female subgroup, the rate of HT was higher in both overall and female patients with effective nAMD than in those with resistant nAMD. Although the relationship between HT and anti-VEGF IVI treatment responsiveness is difficult to interpret, nAMD was reported to be associated with HT, particularly among patients receiving antihypertensive treatment [44]. Since we could not confirm the antihypertensive status of the subjects due to the retrospective nature of the current study, further investigation is needed to clarify the relationship between HT and IVI treatment responsiveness. Another possible limitation is that even though there were no significant differences in the rate of DM between the control subjects and nAMD patients in either the overall group or the female subgroup, the rate of DM was higher in the nAMD patients than in control subjects among the male subgroup. However, this is also understandable, because nAMD is often complicated with DM [45]; thus, it cannot be ruled out that diabetic status may have also affected OS parameters and treatment responsiveness in the male subgroup of the current study.

Nevertheless, no other nAMD study has measured systemic OS parameters and the outcomes of anti-VEGF IVI treatment after one year. This study may thus be considered novel, as it suggests that systemic OS could play a role not only in the development of nAMD but also in its response to anti-VEGF IVI treatment (SAF reference value: 2.4 AU for both). Of course, while OS should be acknowledged as a significant factor in nAMD, it may not be the sole cause of disease development and treatment response.

## Conclusion

The current study revealed that SAF was higher in nAMD patients than in control subjects, and that SAF levels were closely associated with the presence of nAMD and, particularly in male patients, with the number of anti-VEGF IVIs. Specifically, male patients with resistant nAMD had higher SAF levels than male patients with effective nAMD, despite having similar baseline characteristics. These findings were further supported by multiple logistic regression analyses. Given the potential role of AGEs in promoting OS and inflammation, their elevation may contribute to increased anti-VEGF IVI requirements for controlling nAMD activity. From a clinical perspective, assessing systemic OS parameters, such as SAF, could provide valuable insights into both the risk of developing nAMD and the likelihood of requiring more frequent anti-VEGF IVIs.

## Data Availability

The first author, Maiko Abe, had full access to all the data in the study. The data that support the findings of this study are available from the corresponding author, Hiroshi Kunikata, and the first investigator, Maiko Abe, upon reasonable request.

## References

[1] Wong WL, Su X, Li X, Cheung CM, Klein R, Cheng CY, Wong TY. Global prevalence of age-related macular degeneration and disease burden projection for 2020 and 2040: a systematic review and meta-analysis. Lancet Glob Health. 2014;2(2):e106–16. 25104651 10.1016/S2214-109X(13)70145-1

[2] Mitchell P, Liew G, Gopinath B, Wong TY. Age-related macular degeneration. Lancet. 2018;392(10153):1147–59. 30303083 10.1016/S0140-6736(18)31550-2

[3] Brown DM, Kaiser PK, Michels M, Soubrane G, Heier JS, Kim RY, Sy JP, Schneider S, ANCHOR Study Group. Ranibizumab versus verteporfin for neovascular age-related macular degeneration. N Engl J Med. 2006;355(14):1432–44. 17021319 10.1056/NEJMoa062655

[4] Rofagha S, Bhisitkul RB, Boyer DS, Sadda SR, Zhang K, SEVEN-UP Study Group. Seven-year outcomes in ranibizumab-treated patients in ANCHOR, MARINA, and HORIZON: a multicenter cohort study (SEVEN-UP). Ophthalmology. 2013;120(11):2292–9. 23642856 10.1016/j.ophtha.2013.03.046

[5] Traine PG, Pfister IB, Zandi S, Spindler J, Garweg JG. Long-term outcome of intravitreal aflibercept treatment for neovascular age-related macular degeneration using a “treat-and-extend” regimen. Ophthalmol Retina. 2019;3(5):393–9. 31044729 10.1016/j.oret.2019.01.018

[6] Thomsen AK, Fasih-Ahmad S, Sadda S, Sørensen TL. Initial treatment response can predict one-year treatment outcomes in neovascular age-related macular degeneration treated according to the observe-and-plan regimen. Acta Ophthalmol. 2024. 10.1111/aos.16801. 39604206 10.1111/aos.16801

[7] Roddy GW. Metabolic syndrome and the aging retina. Curr Opin Ophthalmol. 2021;32(3):280–7. 33630786 10.1097/ICU.0000000000000747

[8] Chen L, Liu M, Luan Y, Liu Y, Zhang Z, Ma B, Liu X, Liu Y. BMP-6 protects retinal pigment epithelial cells from oxidative stress-induced injury by inhibiting the MAPK signaling pathways. Int J Mol Med. 2018;42(2):1096–105. 29767257 10.3892/ijmm.2018.3675

[9] Nita M, Grzybowski A. Interplay between reactive oxygen species and autophagy in the course of age-related macular degeneration. EXCLI J. 2020;25(19):1353–71. 10.17179/excli2020-2915 PMC7658465 33192217

[10] Yasuda M, Shimura M, Kunikata H, Kanazawa H, Yasuda K, Tanaka Y, Konno H, Takahashi M, et al. Relationship of skin autofluorescence to severity of retinopathy in type 2 diabetes. Curr Eye Res. 2014;40(3):338–45. 24871684 10.3109/02713683.2014.918152

[11] Hashimoto K, Kunikata H, Yasuda M, Ito A, Aizawa N, Sawada S, Kondo K, Satake C, et al. The relationship between advanced glycation end products and ocular circulation in type 2 diabetes. J Diabetes Complications. 2016;30(7):1371–7. 27209548 10.1016/j.jdiacomp.2016.04.024

[12] Himori N, Kunikata H, Kawasaki R, Shiga Y, Omodaka K, Takahashi H, Miyata T, Nakazawa T. The association between skin autofluorescence and mean deviation in patients with open-angle glaucoma. Br J Ophthalmol. 2016;101(2):233–8. 27941048 10.1136/bjophthalmol-2016-309504

[13] Kunikata H, Sato R, Nishiguchi KM, Nakazawa T. Systemic oxidative stress level in patients with central serous chorioretinopathy. Graefes Arch Clin Exp Ophthalmol. 2020;258(7):1575–7. 32266471 10.1007/s00417-020-04664-1

[14] Choi M, Kim SW, Yun C, Oh J. OCT angiography features of neovascularization as predictive factors for frequent recurrence in age-related macular degeneration. Am J Ophthalmol. 2020;213:109–19. 31954711 10.1016/j.ajo.2020.01.012

[15] Khateb S, Chowers I, Grunin M. What can we learn from the surprising insight into the genetic background of age-related macular degeneration and central serous chorioretinopathy? JAMA Ophthalmol. 2023;141(5):457–8. 37079325 10.1001/jamaophthalmol.2023.0927

[16] AnandBabu K, Sen P, Angayarkanni N. Oxidized LDL, homocysteine, homocysteine thiolactone and advanced glycation end products act as pro-oxidant metabolites inducing cytokine release, macrophage infiltration and pro-angiogenic effect in ARPE-19 cells. PLoS ONE. 2019;14(5): e0216899. 31086404 10.1371/journal.pone.0216899 PMC6516731

[17] Matsuura T, Kaneko H, Takayama K, Shibata R, Kataoka K, Ito S, Tsunekawa T, Shimizu H, et al. Diacron reactive oxygen metabolites and biological antioxidant potential tests for patients with age-related macular degeneration. BMC Ophthalmol. 2020;20(1):56. 32070305 10.1186/s12886-020-01334-y PMC7027115

[18] Himori N, Inoue Yanagimachi M, Omodaka K, Shiga Y, Tsuda S, Kunikata H, Nakazawa T. The effect of dietary antioxidant supplementation in patients with glaucoma. Clin Ophthalmol. 2021;15:2293–300. 34113073 10.2147/OPTH.S314288 PMC8183457

[19] Maekawa S, Sato K, Kokubun T, Himori N, Yabana T, Ohno-Oishi M, Shi G, Omodaka K, et al. A plant-derived antioxidant supplement prevents the loss of retinal ganglion cells in the retinas of NMDA-injured mice. Clin Ophthalmol. 2022;16:823–32. 35330750 10.2147/OPTH.S354958 PMC8939866

[20] Sato M, Yasuda M, Takahashi N, Hashimoto K, Himori N, Nakazawa T. Sex differences in the association between systemic oxidative stress status and optic nerve head blood flow in normal-tension glaucoma. PLoS ONE. 2023;18(2): e0282047. 36827337 10.1371/journal.pone.0282047 PMC9955941

[21] Asano Y, Himori N, Kunikata H, Yamazaki M, Shiga Y, Omodaka K, Takahashi H, Nakazawa T. Age- and sex-dependency of the association between systemic antioxidant potential and glaucomatous damage. Sci Rep. 2017;7(1):8032. 28808277 10.1038/s41598-017-08624-4 PMC5556047

[22] Scott E, Zhang QG, Wang R, Vadlamudi R, Brann D. Estrogen neuroprotection and the critical period hypothesis. Front Neuroendocrinol. 2012;33(1):85–104. 22079780 10.1016/j.yfrne.2011.10.001 PMC3288697

[23] Nakazawa T, Takahashi H, Shimura M. Estrogen has a neuroprotective effect on axotomized RGCs through ERK signal transduction pathway. Brain Res. 2006;1093(1):141–9. 16696958 10.1016/j.brainres.2006.03.084

[24] Kitaoka Y, Munemasa Y, Hayashi Y, Kuribayashi J, Koseki N, Kojima K, Kumai T, Ueno S. Axonal protection by 17β-estradiol through thioredoxin-1 in tumor necrosis factor-induced optic neuropathy. Endocrinology. 2011;152(7):2775–85. 21586560 10.1210/en.2011-0046

[25] Ide H, Lu Y, Yu J, China T, Kumamoto T, Koseki T, Yamaguchi R, Muto S, et al. Testosterone promotes DNA damage response under oxidative stress in prostate cancer cell lines. Prostate. 2012;72(13):1407–11. 22290195 10.1002/pros.22492

[26] Son SW, Lee JS, Kim HG, Kim DW, Ahn YC, Son CG. Testosterone depletion increases the susceptibility of brain tissue to oxidative damage in a restraint stress mouse model. J Neurochem. 2016;136(1):106–17. 26385432 10.1111/jnc.13371

[27] Pronsato L, Milanesi L. Effect of testosterone on the regulation of p53 and p66Shc during oxidative stress damage in C2C12 cells. Steroids. 2016;106:41–54. 26703444 10.1016/j.steroids.2015.12.007

[28] Boyer DS, Antoszyk AN, Awh CC, Bhisitkul RB, Shapiro H, Acharya NR, MARINA Study Group. Subgroup analysis of the MARINA study of ranibizumab in neovascular age-related macular degeneration. Ophthalmology. 2007;114(2):246–52. 17270674 10.1016/j.ophtha.2006.10.045

[29] Kaiser PK, Brown DM, Zhang K, Hudson HL, Holz FG, Shapiro H, Schneider S, Acharya NR. Ranibizumab for predominantly classic neovascular age-related macular degeneration: subgroup analysis of first-year ANCHOR results. Am J Ophthalmol. 2007;144(6):850–7. 17949673 10.1016/j.ajo.2007.08.012

[30] Ying GS, Maguire MG, Daniel E, Ferris FL, Jaffe GJ, Grunwald JE, Toth CA, Huang J, et al. Association of baseline characteristics and early vision response with 2-year vision outcomes in the comparison of AMD treatments trials (CATT). Ophthalmology. 2015;122(12):2523-31.e1. 26383996 10.1016/j.ophtha.2015.08.015 PMC4658285

[31] Lee H, Lee M, Kim MA, Chung H, Kim HC. Association of treatment response with quantitative changes in choroidal neovascularization and choroidal vessel in neovascular age-related macular degeneration. Retina. 2020;40(9):1704–18. 31725526 10.1097/IAE.0000000000002678

[32] Han JW, Cho HJ, Kang DH, Jung SH, Park S, Kim JW. Changes in optical coherence tomography angiography and disease activity in type 3 neovascularization after anti-vascular endothelial growth factor treatment. Retina. 2020;40(7):1245–54. 31095063 10.1097/IAE.0000000000002562

[33] Abedi F, Wickremasinghe S, Richardson AJ, Makalic E, Schmidt DF, Sandhu SS, Baird PN, Guymer RH. Variants in the VEGFA gene and treatment outcome after anti-VEGF treatment for neovascular age-related macular degeneration. Ophthalmology. 2013;120(1):115–21. 23149126 10.1016/j.ophtha.2012.10.006

[34] Hagstrom SA, Ying GS, Maguire MG, Martin DF, Gibson J, Lotery A, Chakravarthy U, CATT Research Group, IVAN Study Investigators. VEGFR2 gene polymorphisms and response to anti-vascular endothelial growth factor therapy in age-related macular degeneration. Ophthalmology. 2015;122(8):1563–8. 26028346 10.1016/j.ophtha.2015.04.024 PMC4516643

[35] van Asten F, Rovers MM, Lechanteur YT, Smailhodzic D, Muether PS, Chen J, den Hollander AI, Fauser S. Predicting non-response to ranibizumab in patients with neovascular age-related macular degeneration. Ophthalmic Epidemiol. 2014;21(6):347–55. 25157998 10.3109/09286586.2014.949010

[36] Ioakeimidis N, Gourgouli I, Terentes-Printzios D, Gourgouli DM, Georgakopoulos C, Aznaouridis K, Spai S, Tousoulis D, et al. Aortic stiffness and systemic inflammation changes predict clinical response to intravitreal anti-vascular endothelial growth factor therapy in patients with age-related macular degeneration. J Hum Hypertens. 2023;37(4):273–8. 35474138 10.1038/s41371-022-00689-7

[37] Maguire MG, Bressler SB, Bresskr NM, et al. Risk factors for choroidal neovascularization in the second eye of patients with juxtafoveal or subfoveal choroidal neovascularization secondary to age-related macular degeneration. Arch Ophthalmol. 1997;115(6):741–7. 9194725 10.1001/archopht.1997.01100150743009

[38] Thomsen AK, Steffensen MA, Villarruel Hinnerskov JM, Nielsen AT, Vorum H, Honoré B, Nissen MH, Sørensen TL. Complement proteins and complement regulatory proteins are associated with age-related macular degeneration stage and treatment response. J Neuroinflammation. 2024;21(1):284. 39487449 10.1186/s12974-024-03273-7 PMC11531117

[39] Martinez Villarruel Hinnerskov J, Krogh Nielsen M, Kai Thomsen A, Steffensen MA, Honoré B, Vorum H, Nissen MH, Sørensen TL. Chemokine receptor profile of T cells and progression rate of geographic atrophy secondary to age-related macular degeneration. Invest Ophthalmol Vis Sci. 2024;65(1):5. 38165703 10.1167/iovs.65.1.5 PMC10768715

[40] Rozing MP, Durhuus JA, Krogh Nielsen M, Subhi Y, Kirkwood TB, Westendorp RG, Sørensen TL. Age-related macular degeneration: a two-level model hypothesis. Prog Retin Eye Res. 2019;2020(76): 100825. 10.1016/j.preteyeres.2019.100825 31899290

[41] Nahavandipour A, Krogh Nielsen M, Sørensen TL, Subhi Y. Systemic levels of interleukin-6 in patients with age-related macular degeneration: a systematic review and meta-analysis. Acta Ophthalmol. 2020;98(5):434–44. 32180348 10.1111/aos.14402

[42] Subhi Y, Krogh Nielsen M, Molbech CR, Oishi A, Singh A, Nissen MH, Sørensen TL. Plasma markers of chronic low-grade inflammation in polypoidal choroidal vasculopathy and neovascular age-related macular degeneration. Acta Ophthalmol. 2019;97(1):99–106. 30288946 10.1111/aos.13886

[43] Krogh Nielsen M, Hector SM, Allen K, Subhi Y, Sørensen TL. Altered activation state of circulating neutrophils in patients with neovascular age-related macular degeneration. Immun Ageing. 2017;14:18. 28769990 10.1186/s12979-017-0100-9 PMC5531023

[44] Hyman L, Schachat AP, He Q, Leske MC, Age-Related Macular Degeneration Risk Factors Study Group. Hypertension, cardiovascular disease, and age-related macular degeneration. Arch Ophthalmol. 2000;118(3):351–8. 10721957 10.1001/archopht.118.3.351

[45] Ninomiya T, Kunikata H, Ishikawa M, Nakazawa T. Association between myopia and complications of glaucoma with retinal diseases: investigation of a large medical insurance database. Graefes Arch Clin Exp Ophthalmol. 2023;261(8):2431–3. 36930322 10.1007/s00417-023-06031-2

